# Pre-Clinical Evaluation of a Novel Nanoemulsion-Based Hepatitis B Mucosal Vaccine

**DOI:** 10.1371/journal.pone.0002954

**Published:** 2008-08-13

**Authors:** Paul E. Makidon, Anna U. Bielinska, Shraddha S. Nigavekar, Katarzyna W. Janczak, Jessica Knowlton, Alison J. Scott, Nicholas Mank, Zhengyi Cao, Sivaprakash Rathinavelu, Michael R. Beer, J. Erby Wilkinson, Luz P. Blanco, Jeffrey J. Landers, James R. Baker

**Affiliations:** 1 Michigan Nanotechnology Institute for Medicine and Biological Sciences (M-NIMBS), University of Michigan, Ann Arbor, Michigan, United States of America; 2 Unit for Laboratory Animal Medicine (ULAM), University of Michigan, Ann Arbor, Michigan, United States of America; Centre de Recherche Public-Santé, Luxembourg

## Abstract

**Background:**

Hepatitis B virus infection remains an important global health concern despite the availability of safe and effective prophylactic vaccines. Limitations to these vaccines include requirement for refrigeration and three immunizations thereby restricting use in the developing world. A new nasal hepatitis B vaccine composed of recombinant hepatitis B surface antigen (HBsAg) in a novel nanoemulsion (NE) adjuvant (HBsAg-NE) could be effective with fewer administrations.

**Methodology and Principal Findings:**

Physical characterization indicated that HBsAg-NE consists of uniform lipid droplets (349+/−17 nm) associated with HBsAg through electrostatic and hydrophobic interactions. Immunogenicity of HBsAg-NE vaccine was evaluated in mice, rats and guinea pigs. Animals immunized intranasally developed robust and sustained systemic IgG, mucosal IgA and strong antigen-specific cellular immune responses. Serum IgG reached ≥10^6^ titers and was comparable to intramuscular vaccination with alum-adjuvanted vaccine (HBsAg-Alu). Normalization showed that HBsAg-NE vaccination correlates with a protective immunity equivalent or greater than 1000 IU/ml. Th1 polarized immune response was indicated by IFN-γ and TNF-α cytokine production and elevated levels of IgG_2_ subclass of HBsAg-specific antibodies. The vaccine retains full immunogenicity for a year at 4°C, 6 months at 25°C and 6 weeks at 40°C. Comprehensive pre-clinical toxicology evaluation demonstrated that HBsAg-NE vaccine is safe and well tolerated in multiple animal models.

**Conclusions:**

Our results suggest that needle-free nasal immunization with HBsAg-NE could be a safe and effective hepatitis B vaccine, or provide an alternative booster administration for the parenteral hepatitis B vaccines. This vaccine induces a Th1 associated cellular immunity and also may provide therapeutic benefit to patients with chronic hepatitis B infection who lack cellular immune responses to adequately control viral replication. Long-term stability of this vaccine formulation at elevated temperatures suggests a direct advantage in the field, since potential excursions from cold chain maintenance could be tolerated without a loss in therapeutic efficacy.

## Introduction

Infection with hepatitis B virus (HBV) remains an important global health concern, despite the availability of multiple prophylactic vaccines. The World Health Organization (WHO) estimates that more than 2 billion persons have been infected with the virus. The current prophylactic vaccines require a regimen of three intramuscular (i.m.) injections, have a 10%−15% non-responders rate, and are ineffective for limiting HBV replication in chronic carriers [1], [2], [3]. Large scale vaccination programs are also limited in developing populations due to compliance issues secondary to the three dose vaccination schedule, the requirement for cold storage and the availability of sterile needles [4], [5]. This has limited the use of hepatitis B vaccine in these populations and is partly responsible for 8%−10% of the population in areas of Africa, Asia and South America being chronically infected with HBV [6]. Chronic HBV infection increases the risk of developing liver cirrhosis, hepatocellular carcinoma and other associated complications leading to increased mortality [7]. This suggests the need for a new strategy on hepatitis B vaccination for the developing world.

Hepatitis B surface antigen (HBsAg) is a major structural protein of HBV and is a protective immunogen in experimental animals and in humans [8], [9], [10]. The hepatitis B surface (HBs) proteins are synthesized as large (L), medium (M) and small (S) envelope sub-units, which self assemble into virus-like lipid-anchored particles (about 22 nm in size) [11], [12]. The majority of commercially available recombinant HBsAg vaccines (including Recombivax HB; Merck, and Engerix-B; GSK) consist of yeast derived HBs-S antigen particles adsorbed to an aluminum salt (alum) adjuvant [1], [13]. While alum is generally well tolerated and is considered safe, some adverse effects have been reported [14], [15]. Further, alum has been shown to elicit predominantly a Th2 polarization of immune response, which is associated with cellular immunity that is ineffective at producing CD8 responses to virally infected cells [16]. Since cellular immunity is essential for an efficient response against some infections and elimination of some viral pathogens [17], it would be desirable to develop anti-viral vaccine(s) capable of inducing cell-mediated immunity, in addition to a robust and durable antibody response [17], [18]. Currently available hepatitis B vaccines have comparable thermo-stability profiles requiring unbroken cold chain storage (between 2°C and 8°C) in order to retain potency [19], [20]. The higher costs associated with guaranteed cold chain, from point of manufacture to point of use, also contribute to the inaccessibility of these vaccines. Thus, an efficacious vaccine requiring fewer injections and a less stringent cold storage requirement would directly benefit underserved populations.

Mucosal immunization is an attractive alternative to parenteral vaccines because it can produce both systemic and mucosal immunity and avoids the need for sterile needles [21], [22]. However, development of mucosal vaccines remains limited by lack of effective mucosal adjuvants [23], [24]. Studies have evaluated several potential mucosal adjuvants for hepatitis B vaccines including recombinant cholera toxin (CT) [25], lipid microparticles [26], CpG oligonucleotides [27], [28], cationic particles [29], PLG microspheres [30] or hepatitis B core antigen (HBcAg) [31], [32], [33]. CT has been limited from use in humans due to its potential to cause CNS inflammation. Unfortunately, a CpG-adjuvanted injectable hepatitis B vaccine was recently placed on clinical hold due to inflammatory issues in a patient, further calling into question the safety of pro-inflammatory adjuvants. No other adjuvant, with the exception of HBcAg, has even been tested in clinical trials [34], [35], [36]. This underlines the need for new non-toxic mucosal adjuvants.

This study evaluates the formulation, immunogenicity, thermal stability and pre-clinical safety of a novel mucosal, needle-free hepatitis B vaccine. These vaccine formulations are composed of recombinant HBsAg using nanoemulsions (NEs) (NanoBio Corporation, Ann Arbor, MI) as mucosal adjuvants. NEs are nanoscale (<400 nm) emulsions formulated with surfactants, distilled water, refined soybean oil and ethanol as a solvent. Initially developed as broad-spectrum antimicrobial agents [37], [38], [39], NEs proved effective as mucosal adjuvants for a range of antigens from whole influenza and vaccinia viruses to recombinant anthrax protective antigen and HIV gp120 [40], [41], [42], [43]. In animals, these experimental NE-based vaccines do not produce local inflammation as is observed with alum-based vaccines [15]. However, these formulations are effective in inducing Th1 biased immune response and cellular immunity [41], and remain stable for at least three years at 25°C (Hamouda T., NanoBio, personal communication). These inherent advantages of NE provided a rationale for evaluating it as a mucosal adjuvant in a candidate nasal vaccine for prevention of hepatitis B infection.

## Results

### Characterization of vaccine formulation

The candidate hepatitis B vaccine was formulated from two components; recombinant HBsAg and NE (HBsAg-NE). The formulation was characterized by evaluating the stability of its components, as well as the physical interaction of the antigen with NE.

To characterize NE stability, emulsion droplet size was analyzed in a broad range of concentrations (1% to 40%) both alone and mixed with either low (0.5 mg/ml) or high (2.5 mg/ml) concentrations of HBsAg. The effect of temperature on the mixing of antigen and NE of was evaluated by incubating each of the formulations at either 4°C, 25°C, or 40°C for 72 hours. The lipid droplet size was stable and uniform in both concentrations of antigen (the average size for all conditions calculated as 349±17 nm), and droplet size of the mixture was not altered by either temperature or NE concentration (**Table 1**).

**Table 1:** Consistency of nanoemulsion particle size.

Particle Size				
	Temperature Condition (°C)			
Sample description	Fresh	4	25	40
1% W_80_5EC	355 (+/−130)	385 (+/−141)	327 (+/−168)	353 (+/−201)
20% W_80_5EC	368 (+/−255)	328 (+/−159)	350 (+/−166)	337 (+/−166)
40% W_80_5EC	331 (+/−154)	373 (+/−221)	322 (+/−178)	340 (+/−153)
20% W_80_5EC+0.5 mg/ml HBsAg	373 (+/−229)	325 (+/−142)	354 (+/−193)	347 (+/−200)
20% W_80_5EC+2.5 mg/ml HBsAg	341 (+/−143)	348 (+/−177)	361 (+/−227)	347 (+/−232)

Average particle size 349 (+/−17); Mean (+/−SD).

**Figure 1:**
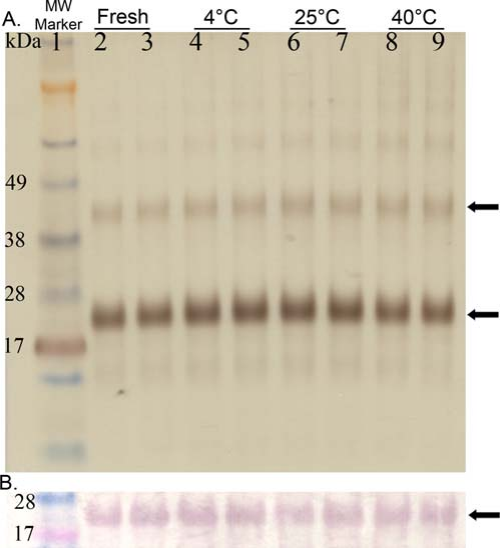
Stability of HBsAg. Silver stained SDS-PAGE: Analysis of freshly prepared vs. 72 hour incubated HBs antigen mixed with 20% NE (A). Conditions of the incubation are specified above the gel. HBsAg monomer and dimer are indicated with arrows at 24 kDa and 48 kDa bands. Western blot: The protein configuration is identical to as described for SDS-PAGE (B). HBsAg is detected using a polyclonal anti-HBsAg antibody for all incubation conditions.

HBsAg integrity in the emulsion was evaluated using SDS-PAGE and Western blot (**Figures 1A and B**). NE also did not interfere with the electrophoresis or immunoblotting procedures. After treatment with SDS, HBsAg protein migrated as a band that corresponded to HBsAg monomer (Mw≈24 kDa) with a minor fraction at twice this molecular weight representing dimer, and this pattern was not altered by prior mixing in NE. In addition, antigenic recognition was retained in HBsAg mixed in NE as identified in Western Blots using a polyclonal goat antiserum raised to native HBsAg (**Figure 1B**). No degradation products of HBsAg were detected in either analysis and was no significant aggregation appeared to occur during mixing or incubation with NE.

**Figure 2:**
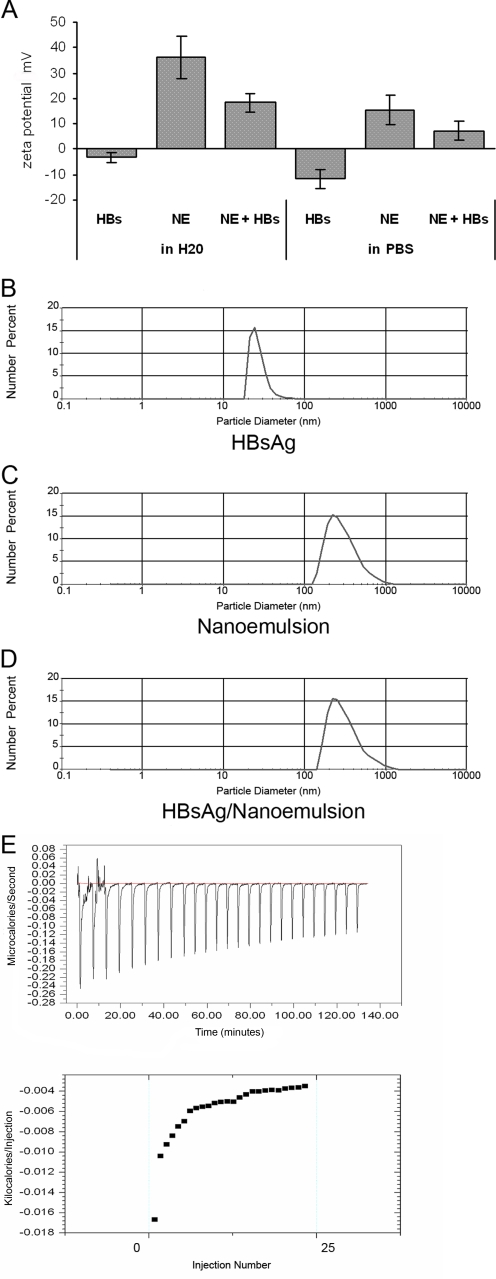
HBsAg interaction with NE droplets. Zeta potential: (A) The zeta potential was measured for HBsAg (2.5 mg/ml), 20% NE and mixture of HBsAg with NE (HBsAg-NE) in water and PBS. Surface charge is reported in mV units. Particle sizing: Size distribution was measured using a laser diffraction particle-sizer. Analysis of HBsAg alone (B), NE alone (C), and NE with 10 μg/ml of HBsAg (D). Data was processed and analyzed using Fraunhofer optical modeling and number weighted averaging (number %). Single population intensity peaks indicate monodisperse populations of HBsAg (28 nm), NE (349 nm), and HBsAg-NE (335 nm). Calorimetric titration of HBsAg with NE: 25 injections of 1% NE (10 μl/injection) were introduced into a sample cell containing HBsAg (600 μg/ml in PBS) at 30°C. The upper panel shows differences between the sample and reference cell containing PBS. The lower panel shows enthalpy per injection of NE injected versus injection number. An exothermic reaction was measured following the addition of nanoemulsion to an antigen solution. Addition of nanoemulsion to the antigen solution became less energetic as the total concentration of nanoemulsion increased, suggesting that the antigen was being depleted. ΔCp was calculated to be −1.44 [77].

The surface charge of the vaccine formulation was determined by measuring the zeta potential and was compared to NE and HBsAg solutions with deionized water and PBS buffer used as diluents. In either diluent, HBsAg had negative zeta potential as has been previously reported in literature (**Figure 2A**) [44]. In contrast, there was a decrease in the positive zeta potential of the NE after mixing with the HBsAg. This suggests an electrostatic association between the negatively charged HBsAg particles and cationic CPC-containing emulsion [45]. The drop in charge of the emulsion was more pronounced when the HBsAg/NE formulation was made with deionized water as compared to buffers such as PBS (**Figure 2**).

The interaction of HBsAg with NE was further examined using laser diffraction particle sizing and isothermal titration calorimetry (ITC). Two independent and differently sized peaks for NE and HBsAg were observed before mixing, however after formulation only a single peak was detected with a dynamic diameter of ∼300 nm (**Figures 2B, C, and D** **and** **Table 1**). The absence of two separate peaks again indicated an association between the lipid phase and HBsAg protein, and suggested that no significant fraction of the antigen remained independent from the lipid in the aqueous phase of NE. Thermodynamic analysis of the interaction between the HBsAg and the NE using ITC showed a spontaneous exothermic reaction with a calculated change in heat capacity of binding (ΔCp) of −1.44 indicating an energetically favorable interaction (**Figure 2E**).

### Optimization the immunogenicity of the nasal HBsAg-NE vaccine

**Figure 3:**
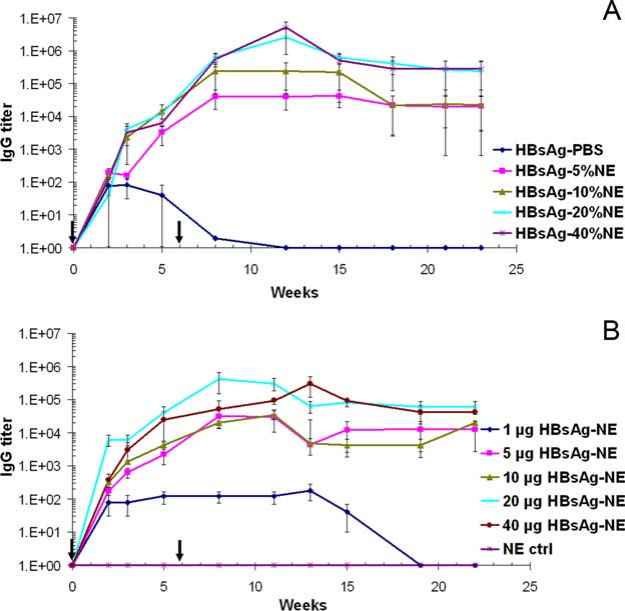
Development of IgG response in serum. Effect of NE adjuvant: Mice were immunized intranasally with HBsAg-NE consisting of 20 μg of HBsAg mixed with 0% to 40% concentrations of NE (A). Significant statistical difference with *p value<0.05* was observed between all HBsAg-NE formulations and HBsAg-PBS vaccine. Antigen dose escalation: Mice were immunized intranasally with 1 μg to 40 μg HBsAg mixed with 20% NE (B). A significant statistical difference with *p value<0.05* was observed between all 5 μg to 40 μg HBsAg-NE formulations and 1 μg HBsAg-NE vaccine. Serum anti-HBsAg IgG antibody concentrations are presented as mean of endpoint titers in individual sera +/− SEM. * indicates a statistical difference (*p value<0.05*) in the anti-HBsAg IgG titers. Arrows indicate vaccine administration.

Immunogenicity of the HBsAg-NE vaccine formulation was optimized by conducting *in vivo* adjuvant and antigen dose escalation studies. To optimize the NE concentration for the vaccine, CD-1 mice were intranasally (i.n.) immunized with 20 μg of HBsAg mixed with a range of NE concentrations from 5% to 40% using antigen in PBS as a control. After a single immunization with 20 μg of HBsAg in either 10%, 20% or 40% NE, similar end-point serum anti-HBsAg IgG titers averaging over 10^4^ were achieved **(** **Figure 3A** **)**. In contrast, significantly lower serum titers (<10^2^) were generated after immunization with 5% NE and low, inconsistent antibody responses were detected in mice nasally vaccinated with HBsAg in PBS **(** **Figure 3A** **)**. Booster immunization at six weeks caused the serum anti-HBsAg IgG titers to increase over 10 fold in all groups except in the animal immunized with HBsAg in PBS where no effect was observed. The highest anti-HBsAg antibody endpoint titers, exceeded 10^6^ at 6 to 8 weeks after boost, were achieved when the animals were vaccinated with either 20% or 40% NE. The HBsAg-NE vaccine also produced persistent antibody responses with serum anti-HBsAg IgG titers of 10^4^–10^5^ at 6 months after initial vaccination regardless of the concentration of NE used for vaccination. Thus, the lowest optimal NE concentration was determined to be 20%.

To optimize the antigen concentration in the NE vaccine, mice were i.n. immunized with 1 μg, 5 μg 20 μg and 40 μg of HBsAg mixed with 20% NE **(** **Figure 3B** **)**. After a single vaccination anti IgG HBsAg antibody responses showed a dose dependent relationship with highest titers in the 20 μg HBsAg-NE group and significantly weaker antibody responses in mice vaccinated with 1 μg of HBsAg. After a second immunization at six weeks, the anti-HBsAg IgG titers increased approximately 10 fold exceeding 10^4^, except in animals immunized with 1 μg HBsAg in NE. Intranasal immunizations with equivalent amounts of HBsAg mixed in PBS again produced only sporadic and weak antibody responses with titers less than 10^2^ (data not shown). This indicated that 20 μg of HBsAg appeared in the optimal antigen dose.

### Immunogenicity of HBsAg-NE immunization

**Figure 4:**
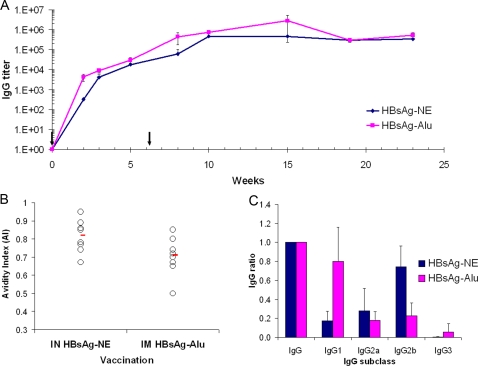
Comparison of mucosal NE-based with conventional aluminum-based injectible HBsAg vaccine. Time course of antibody response: Mice were immunized with 20 μg HBsAg. Antigen was mixed with 20% NE for intranasal administration (HBsAg-NE), or adsorbed on aluminum hydroxide (HBsAg-Alu) for intramuscular injections (A). Serum anti-HBsAg IgG antibody concentrations are presented as mean of endpoint titers in individual sera +/− SEM. * indicates a statistical difference (*p value<0.05*) in the anti-HBsAg IgG titers. Arrows indicate vaccine administration. Avidity of anti-HBsAg IgG: Analysis of sera from mice immunized i.n. with HBsAg-NE and with i.m. injections of HBsAg-Alu vaccines (B). Avidity indexes (AI) were assessed using 1.5 M NaSCN as a discriminating salt concentration. Each circle represents individual AI and lines indicate mean AI value for each group obtained in two independent assays. Serum anti-HBsAg IgG subclass: Anti-HBsAg IgG subclass pattern in mice immunized nasally with HBsAg-NE and injected i.m. with HBsAg-Alu vaccine (C). Analysis of sera collected at 22 weeks after initial immunization. The results are presented as ratio of the specific subclass IgG to the overall IgG titer. * indicates statistical difference (*p value<0.05*) between NE-based and alum-based immunizations.

The humoral and cell-mediated immune responses to the optimized HBsAg-NE vaccine were characterized *in vivo* in mice. Intranasal vaccination with either 20 μg HBsAg-20% NE or i.m. injection of 20 μg HBsAg-Alu resulted in comparable, high levels of anti-HBsAg serum IgG antibodies reaching 10^5^ to 10^6^ titers within 8 weeks after primary vaccination (**Figure 4A**). HBsAg-Alu induced a slightly more rapid response, producing higher IgG titers than HBsAg-NE at 2 weeks after primary vaccination (*p value<0.05*), but this difference disappeared after a single booster immunization. Both HBsAg-NE and HBsAg-Alu vaccines produced equivalent, durable immune responses with serum anti-HBsAg IgG end point titers of 10^4^ to 10^5^ being maintained up to 6 months after vaccination. Nasal vaccination with HBsAg-NE elicited serum titers in mice that when normalized with standardized human anti-HBsAg serum indicated an antibody index ≥1000 IU/ml (data not shown). This index is compatible with protective immunity in humans.

Analysis of serum IgG anti-HBsAg avidity at 23 weeks indicated significantly higher antibody avidity in HBsAg-NE immunized animals as compared to IgG from HBsAg-Alu vaccinated mice (*p value = 0.034*) (**Figure 4B**). While the overall titers were equivalent, analysis of serum IgG subclass indicated that i.n. HBsAg-NE vaccination produced anti-HBsAg IgG with a prevalence of IgG2b (and IgG2a) over IgG1 subclass antibodies, while consistent with previous reports [46], [47] the HBsAg-Alu vaccine produced mainly IgG1 subclass antibodies (**Figure 4C**). This suggests a Th1 response to the NE-based vaccine vs. the traditional Th2 response associated with alum. Immunization with HBsAg-NE composed of *adw* serotype surface antigen also produced cross-reacting IgG antibodies against the heterologous *ayw* serotype (data not shown).

**Figure 5:**
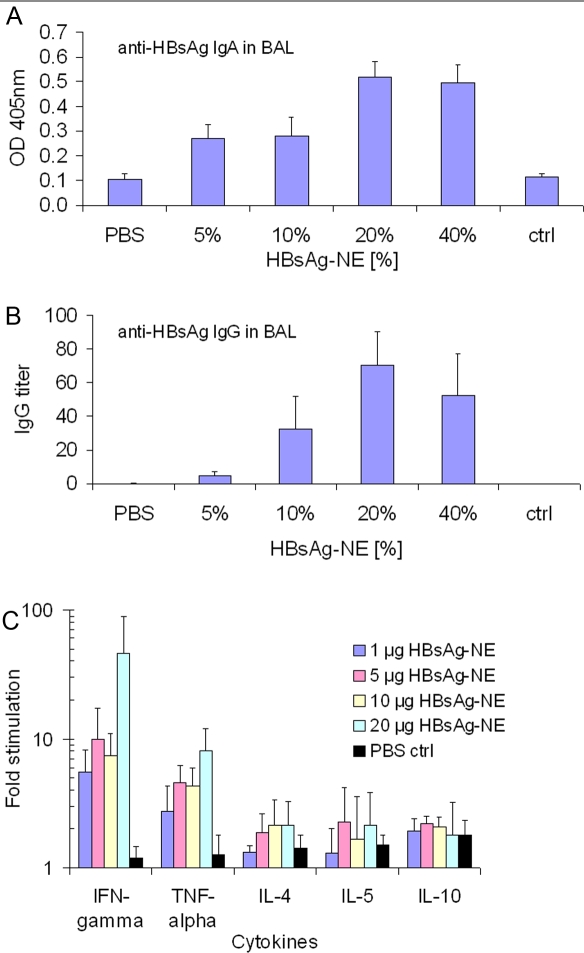
Characterization of immune response mucosal and cellular responses to HBsAg-NE. Mucosal antibody measurements were performed in BAL fluids obtained 23 weeks after i.n immunization with HBsAg-NE vaccines. IgA detection: The anti-HBsAg IgA detection was performed with 1:2 dilutions of BAL (A). Results are presented as mean values of OD at 405 nm +/−SEM. IgG detection: The anti-HBsAg IgG antibody concentrations are presented as end point titers (B). Antigen-specific cytokine expression. Pattern of Th1 (IFN-γ and TNF-α) and Th2 (IL-4, IL-5 and IL-10) cytokine expression in vitro in splenocytes from mice intranasally immunized with HBsAg-NE (C). Splenocytes were obtained at 23 weeks after initial immunization from mice with HBsAg-20% NE. Results are presented as fold increase of the cytokine production over levels detected in non-stimulated splenocyte cultures.

Mucosal immune responses were characterized in bronchioalveolar lavage (BAL) fluid of immunized animals. HBsAg specific IgA and IgG antibodies were detected in BAL samples obtained at the conclusion of the study (23 weeks after initial immunization) from mice immunized intranasally with HBsAg-NE (**Figure 5A and B**, **respectively**). These animals also had detectable serum levels of IgA anti-HBsAg (data not shown). No anti-HBsAg antibodies were detected in BALs or serum in mice immunized with antigen administered in PBS or in intramuscularly immunized mice despite high serum titers.

HBsAg specific cellular responses were characterized in splenocytes of immunized outbred CD-1 mice obtained at 18 weeks after last immunization. The cells were stimulated with HBsAg and then evaluated for specific cytokine production (**Figure 5C**). The cytokine expression pattern included high production of the Th1-type cytokines IFN-γ and TNF-α (ranging from 5 to 40 fold) and lower increases (≤2 fold) in the expression of Th2-type cytokines IL-4, IL-5 and IL-10. This pattern of expression suggested a Th1 bias of cell-mediated response.

**Figure 6:**
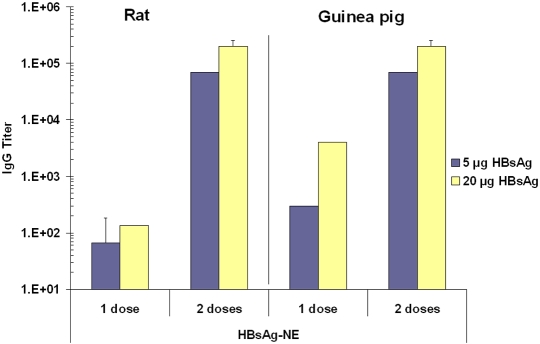
Immunogenicity in rats and guinea pigs. Rats and guinea pigs were immunized intranasally with either 5 μg or 20 μg HBsAg mixed with 20% NE. Serum anti-HBsAg IgG antibody concentrations are presented as mean of endpoint titers in individual sera±SEM. Data represents anti-HBsAg end titers measured 3 weeks after a single dose (1 dose) vaccination and after two vaccinations (2 doses) measured at 7 weeks.

The serum IgG response elicited by HBsAg-NE vaccine was also studied in two alternative rodent species to ensure that the immunization effect was not species specific. Rats and guinea pigs were immunized with 5 μg and 20 μg doses of HBsAg mixed with 20% NE (**Figure 6**). After a single vaccination, animals showed a dose dependent response with the highest IgG antibody titers in the 20 μg HBsAg-NE group. After a second administration at five weeks, the anti-HBsAg IgG titers increased up to 100 and 1000 fold surpassing 10^5^ titers in both species. Thus, the HBsAg-NE vaccine appeared immunogenic in all three animal species tested.

### Thermal Stability of HBsAg-NE vaccine

The optimized formulation of HBsAg-NE was evaluated for thermal stability at three test temperatures. The vaccine product was mixed to contain 2.5 mg/ml HBsAg in 20% NE (to obtain a single dose of 20 μg of antigen in a final volume of 8 μl) and aliquoted into capped glass vials for storage at 4±2°C, 25±2°C or 40±2°C. At 6 weeks, 3 months, 6 months and a year after the start of the stability study aliquots of the formulation were evaluated for physical stability *in vitro* and immunogenicity *in vivo*.

**Figure 7:**
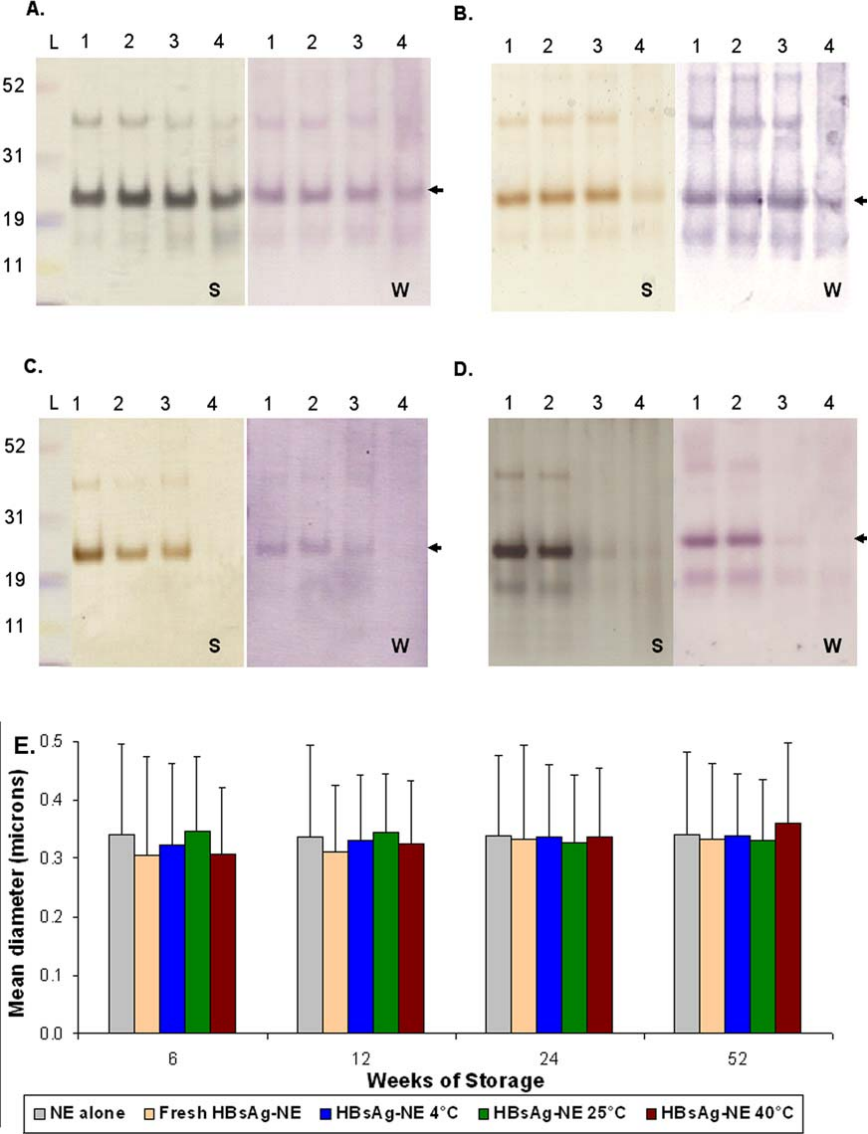
In vitro analyses of HBsAg-NE stability. The comparison of HBsAg-NE stored at test temperature conditions to freshly prepared formulation using SDS-PAGE followed by silver staining (S) or Western immunoblotting (W) is shown. Lanes are labeled according to sample storage conditions as follows- 1: fresh, 2: 4°C, 3: 25°C and 4: 40°C. Samples were stored for (A) 6 weeks, (B) 6 months (24 weeks), or (C) 1 year (52 weeks) at the three test temperatures. Each lane contains 0.5 μg of antigen. Arrow indicates the HBsAg major band. (D) Particle size comparison of NE alone to freshly mixed HBsAg-NE and formulation stored up to a year. Mean diameter of particles is shown in microns +/− SD.

HBsAg stability in vaccine samples was analyzed by SDS-PAGE with silver staining and antigenicity evaluated with Western blots (**Figure 7**) with the stored samples compared to freshly mixed vaccine at each time point. The protein stains and Western Blots of HBsAg at 6 weeks and 3 months were not different from fresh material and there were no low molecular weight degradation products appreciable at these or any later time points (**Figure 7A and B**). After 6 months of storage (**Figure 7B**), however, the major HBsAg band was not detectable in the 40°C by silver staining or immunoblotting, whereas both 4°C and 25°C stored products were still comparable to freshly mixed vaccine. After 1 year of storage (**Figure 7C**), the 25°C sample was also degraded, while the 4°C stored formulation was intact and comparable to freshly mixed vaccine. The stability of the NE also was evaluated by particle size characterization (**Figure 7D**). The mean diameter (±SD) of freshly mixed HBsAg-NE samples was 0.323±0.016 μ, and there were no significant differences between NE particle sizes of fresh and stored HBsAg-NE samples at any temperature or time point.

**Figure 8:**
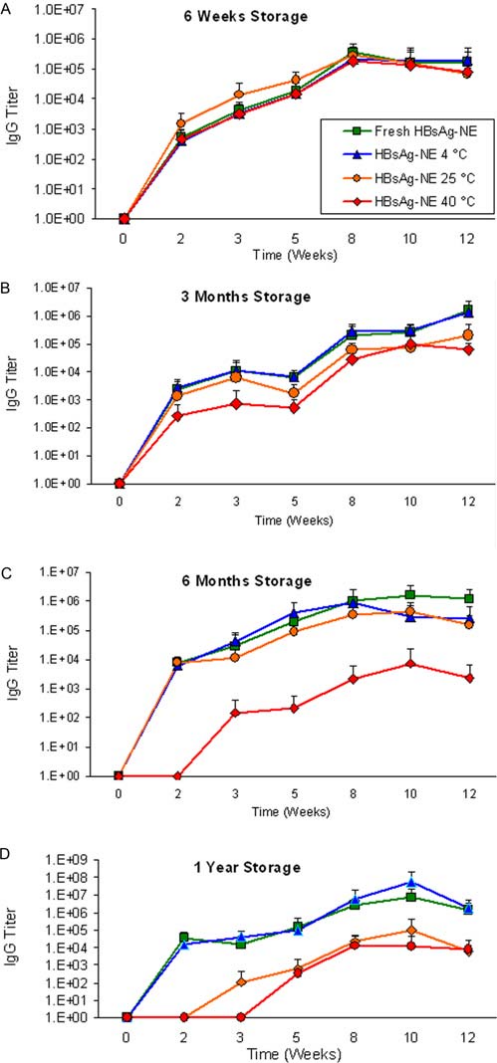
In vivo analyses of HBsAg-NE stability. HBsAg specific antibody responses to freshly prepared HBsAg-NE or HBsAg-NE stored under real-time (4°C), accelerated (25°C) and stressed (40°C) temperature conditions are depicted. CD-1 mice were vaccinated with either freshly prepared or stored HBsAg-NE and boosted at 6 weeks. Serum anti-HBsAg IgG antibody concentrations are presented as a mean of endpoint titers in individual sera +/− SD. Comparison of serum IgG elicited by freshly prepared HBsAg-NE to formulation stored for (A) 6 weeks, (B) 3 months, (C) 6 months or (D) 1 year at indicated temperatures. * indicates a statistical difference (*p value<0.05*) in the anti-HBsAg IgG titers between freshly mixed and stored formulation. Arrows indicate vaccine administration.

Immunogenicity of the vaccine in CD-1 mice was tested at each time point and storage temperature. Mice were immunized then boosted at six weeks post-vaccination, and anti-HBsAg serum IgG responses were determined at 2, 3, 5, 8, 10 and 12 weeks after primary vaccination. There were no significant differences in serum IgG titers elicited by HBsAg-NE vaccine stored at any temperature up to 3 months (**Figure 8A and B**). At 6 months of storage, HBsAg-NE stored at 40°C could elicit and boost HBsAg-specific antibodies, but at a significantly decreased titer when compared to freshly mixed vaccine, while 4°C and 25°C stored vaccines retained complete immunogenicity (**Figure 8C**). After 1 year of storage, 25°C stored HBsAg-NE elicited decreased serum IgG while the 4°C and 25°C stored vaccines again retained complete immunogenicity (**Figure 8D**). This indicated that the vaccine retained immunogenicity for 3 months at 40°C and 6 months at 25°C.

### Evaluation of the safety of NE adjuvant and HBsAg-NE vaccine

**Figure 9:**
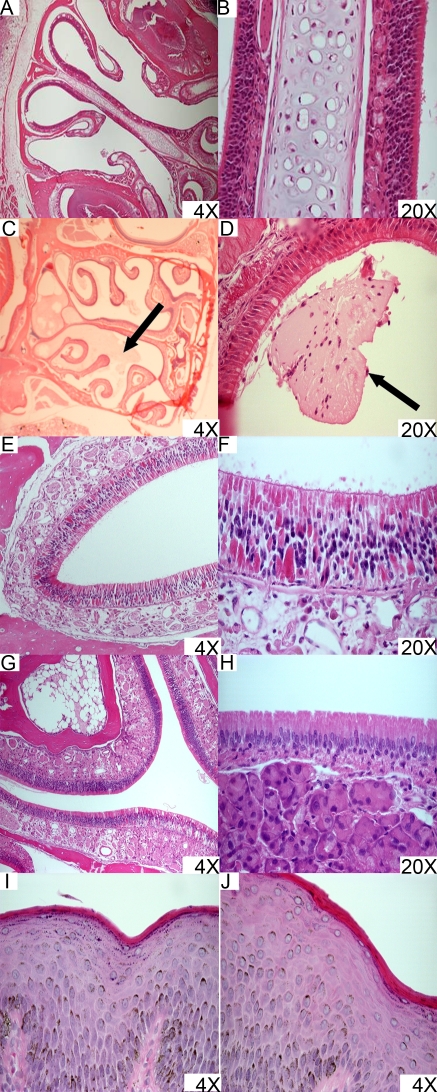
Histopathological analysis of nasal tissue exposed to NE adjuvant or HBsAg-NE. Mice: CD-1 mice were vaccinated with HBsAg-NE and primed at 2 weeks. (A–B) Photomicrographs of H&E stained nasal epithelium collected from mice 14 days following the boost vaccination. Only normal tissue architecture was recorded which indicates a lack of (sub) chronic cytotoxicity or inflammation. (C) Nasal epithelium collected 24 hours following boost vaccination with HBsAg-NE scored as a +1 grade change according to methodology as described is shown as an example of architectural change. The arrow indicates a single microscopic focus of accumulation of mucoid material and debris in the nasal passages. However, no evidence of epithelial necrosis or inflammatory infiltration of the nasal epithelium is detected. (D) Nasal epithelium collected 24 hours following boost vaccination with HBsAg-NE scored as a +2 grade. The arrow indicates a single microscopic focus of accumulation of mucoid material and debris in the nasal passages in the absence of inflammatory changes. Architectural change demonstrated in C and D are considered incidental and can be observed in non-vaccinated mice. Rats and guinea pigs: Nasal epithelium was collected 14 days following final boost vaccination from rats (E–F) and guinea pigs (G–H) treated a total of 3 doses of HBsAg-NE administered 14 days apart. Normal tissue architecture is observed suggesting lack of toxicity or inflammation. Dogs: Nasal biopsies were collected 24 hours following the final dose in dogs treated with a total of three doses of NE adjuvant. NE was delivered using a Pfeiffer multidose wide angle sprayer pump in 200 μl/dose (I) or 400 μl/dose (J). No evidence of inflammation or cytotoxicity was detected.

Comprehensive evaluation of acute and (sub) chronic toxic effect of NE and HBsAg-NE formulations was performed in rodent models and in dogs. Multiple intranasal dose studies (ranging from 2 to 7 total doses as shown in **Table 2**) for NE adjuvant or HBsAg-NE were conducted. No statistically significant changes in subcutaneous temperature or body weight were observed in these species as compared to non-treated control groups (data not shown). Likewise, no observable changes in activity or appetite were noted throughout the study. Hematological and serum biochemical results in rats, guinea pigs, and dogs were within normal physiological range (**Table 2**). No lesions were reported in highly perfused organs including the olfactory bulb and frontal lobe of the brain. Cytotoxicity was not observed in nasal epithelium and other exposed tissues. The only histological lesion noted was the accumulation of amorphous material that sometimes contained cellular debris from sloughed nasal epithelial cells. None of the lesions were of clinical significance (**Table 2** **and** **Figure 9**). For further analysis, serum was collected in mice 24 hours following i.n. vaccination with NE-HBsAg and was evaluated for the presence of IL-1α. IL-1β, IL-2, IL-4, IL-5, IL-6, IL-7, IL-9, IL-10, IL-12, IL-13, IL-15, IL-17, G-CSF, GM-CSF, IFN-γ, IP-10, KC, MCP-1, MIP-1α, RANTES, TNF-α using a LINCOplex® Mouse Cytokine/Chemokine kit. Serum concentrations of IL-6 significantly exceed those of the non-treated controls (933 pg/ml NE-HBsAg vs. 174 pg/ml for PBS control [*p value* = 0.053]). The production of IL-6, however, occurs in the absence of neutrophilic, macrophagic, or other inflammatory cellular infiltration in directly exposed nasal epithelium (**Figure 9**). Overall, both NE and HBsAg-NE were safe and well tolerated by all animal species tested. Approximately 5% of mice developed nasal obstruction with the emulsion, but this was not observed in larger animals and appeared to be related to the unique nasal anatomy of the mouse.

**Table 2:** Pre-clinical toxicology evaluation.

Species/Strain^a^	Treatment		Number of Dose	Dose Volume (ul)	Group Average Histopathological Score^b^				Metabolic Analysis^d^
	NE (%)	HBsAg (ug)			Nasal	Pulmon.	Brain	Other^c^	
Mouse/CD-1	0	20	2^e^	10	0	0	0	0	n/a
	1	20	2^e^	10	0	0	0	0	n/a
	5	20	2^e^	10	1.0±0.9	0	0	0	n/a
	10	20	2^e^	10	0.7±1.1	0	0	0	n/a
	20	20	2^e^	10	1.4±1.3	0	0	0	n/a
	20	0	2^e^	10	1.1±1.7	0	0	0	n/a
Mouse/BALB/c	20	0	4^f^	6	1.2±0.4	0	0	0	n/a
	20	0	7^g^	6	2.0±1.0	0	0	0	n/a
Rat/Wistar	20	32	3^h^	20	0	0	0	0	Normal
Guinea Pig/Hartley	20	32	3^h^	20	0	0	0	0	Normal
Canine/Beagle	20	0	3^e^	200	0	n/a	n/a	n/a	Normal
	20	0	3^e^	400	0	n/a	n/a	n/a	Normal

a - The number of animals used for analysis: CD-1 (n = 10), BALB/c, Wistar rats and Hartley guinea pigs (n = 5) and Beagles (n = 1) per group.

b - Histological lesions were evaluated on a scale from 0 to 10 with +1 single microscopic focus, +2 at least 2 microscopic foci, +3 more than 3 foci or multiple locally extensive areas of pathology, +4 to +6 were associated increasing severity and more extensive distribution (these lesions could be associated with morbidity), +7 and above had increasing degrees of inflammation (+10 associated with mortality).

c - Other tissues evaluated include heart, liver, kidneys, spleen, esophagus, trachea, stomach, intestines, pancreas, and adrenals.

d - Metabolic analysis evaluated by standard biochemical serum profile analysis on a IDEXX Vet Test Analyzer™ and performed at the Animal Diagnostic Laboratory through the Unit for Laboratory Animal Medicine at the University of Michigan. Normal indicates all analytes fell within normal expected distributions per species.

e - Administered every 2 weeks.

f - Administered every 15 minutes.

g - Administered every 4 hours.

h - Administered every 4 weeks.

## Discussion

The commercial hepatitis B vaccines are a remarkable triumph in disease prevention. As one of the first recombinant biological products used as a vaccine, these formulations have a 20 year record of efficacy in preventing hepatitis B and have excellent safety with local reactions from the alum adjuvant being the predominant adverse event [1], [2], [48], [49]. However, hepatitis B remains a significant health issue, especially in the developing world. While efforts of local producers have greatly reduced the cost of the vaccine, the requirement for sterile needles and syringes for administration, and a refrigerated cold chain have conspired to prevent vaccination of many at-risk populations. Because of these issues there is an ongoing interest in producing needle-free and thermally stable hepatitis B vaccines with similar safety and efficacy to the current commercial alum-based vaccines [19], [20], [50].

This study documents the immunogenicity of a novel, mucosal hepatitis B vaccine that is based on a mixture of recombinant HBsAg and nanoemulsion adjuvant. A single nasal immunization of the HBsAg-NE mixture produces a rapid induction of serum anti-HBsAg IgG, which is comparable to that achieved with i.m. vaccination using aluminum salt-based vaccine. Serum IgG responses could be boosted and the titers persisted for the 23 weeks duration of these studies. Normalization carried out by comparison to a standardized human anti-HBsAg serum indicated that anti-HBsAg antibody titers in mice immunized with a nasal HBsAg-NE vaccine corresponded to a greater than 1,000 mIU/ml HBsAg IgG concentration in humans which are considered to be seroprotective against HBV infection [2], [51]. There was also evidence suggesting affinity maturation in the antibody response as serum IgG from animals vaccinated with HBsAg-NE indicated that their avidity matured over time to achieve higher values at 23 weeks than at 5 weeks after vaccination (data not shown). This is important since functional antibody maturation is considered a significant correlate for the protective efficacy of vaccines [52], [53]. The cross-reactive nature of IgG antibodies against the heterologous *ayw* serotype supports the idea that immunization with one of HBsAg serotypes may produce IgG response broadly reactive with HBsAg epitope variants. This could be of importance for the protective immunity against various serotypes of HBV.

In these studies nasal immunization with HBsAg-NE also induced significant mucosal immunity as documented by IgA and IgG detected in BAL fluids, although the role of mucosal IgA in protecting from a blood-borne pathogen like HBV is not clear. Mucosal immunization with HBsAg-NE also induced antigen-specific T cell responses similar to those reported by our group when using NE as a mucosal adjuvant for gp120, rPA, and vaccinia virus in various mouse strains [40], [41], [54]. *In vitro* stimulation of splenocytes harvested from vaccinated mice with HBsAg resulted in a cytokine response characterized by significant secretion of hallmark Th1 type cytokines such as IFN-γ and TNF-α, while Th2 type cytokines IL-4, IL-5 and IL-10 showed no antigen-specific response [55], [56], [57]. In addition to enhancing the magnitude of antibody response, nanoemulsion adjuvant clearly had an effect on the pattern of IgG isotypes, as indicated by prevalence of IgG2 over IgG1 subclass in contrast to vaccination with HBsAg-Alu which produced overwhelming titers of IgG1 antibodies [32], [58]. Prevalence of IgG2b in the overall IgG response provided additional confirmation of a Th1 bias in cellular immunity produced by HBsAg-NE vaccination. However, it is important to note that IgG1 still remained at significant titers, which may suggest co-activation of both Th1 and Th2 immune elements [59]. Although this effect resembles immunostimulation with MPLA, CpG or the other TLR agonists, the lack of any pro-inflammatory compound in the NE and the absence of local nasal inflammation or irritation from the HBsAg-NE argues against activation of these pathways [60], [61]. In general, pro-inflammatory cytokines were not detected systemically in serum with the exception of elevated levels IL-6 up to 24 hours following intranasal treatment with NE-HBsAg. Although the role of IL-6 in mucosal environment is fully understood, it has been shown to be play a variety of regulatory functions in development of immune response [62], [63], [64]

These studies are significant for a number of reasons. This vaccination produced immunity to HBsAg compatible with aluminum salt-adjuvanted vaccines, but without the need for injection or an inflammatory adjuvant. The ability to produce this level of serum antibodies with nasal immunization is also unique, and shows the potential of this methodology. In addition, the simple approach to formulation of this vaccine makes it suitable to be produced without special equipment. These characteristics should facilitate the use of this vaccine in developing regions of the world. The other advantage of this formulation is its stability. Two mechanisms (electrostatic and hydrophobic interactions) of antigen-lipid association can be documented. The exothermic reaction upon mixing these components also suggests that the mixture is more stable than protein in aqueous solution. The association of the antigen complex with NE through hydrophobic mechanisms seems plausible given the negative change in heat capacity (Cp) and because HBsAg particles contain ∼20% host-cell derived lipids [65], [66], [67]. Laser diffraction particle sizing and zeta potential measurement indicate that cationic lipid-phase NE droplets associate with HBsAg and remain uniform in size and stable over a broad range of concentrations and temperature conditions. This indicates that the physical association of HBsAg with the lipid phase of NE provides stability to the antigen as well as contributing to the adjuvant capability of NE. Because maintaining cold chain conditions of 4°C to 8°C during inventory, storage and transport contributes significantly to the cost of the currently licensed hepatitis B vaccines, this stability is significant. Since the HBsAg-NE vaccine retained immunogenicity up to 6 months at 25°C and 3 months at 40°C, the vaccine might not require refrigeration for the final stages of distribution. At the least, it indicates that accidental breaks in the cold chain, as often occurs in developing countries, would not necessitate the destruction of the vaccine. Also, the decreases in immunogenicity were readily detected by *in vitro* analyses (**Figure 7B and 7C**), allowing for easy evaluation of vaccine stocks.

Adjuvants have been traditionally developed from pro-inflammatory substances, such as a toxin or microbiological component, found to trigger signaling pathways and cytokine production [68]. Also, enterotoxin-based adjuvants, such as cholera toxin, have been associated with inducing inflammation in the nasal mucosa and with production of the inflammatory cytokines and transport of the vaccine along olfactory neurons into the olfactory bulbs, [69]. Some patients treated with a flu vaccine based on one of these toxins (Nasalflu, Berna Biotech), developed Bell's palsy [70] presumably due to the vaccine in the olfactory bulb. This finding led to Nasalflu being withdrawn, and it is unclear whether other mucosal vaccines using this approach will be acceptable for humans. Our studies indicate no significant inflammation in HBsAg-NE treated animals and there was no evidence of the vaccine in the olfactory bulb. These finds are encouraging and suggest that successfully nasal mucosal adjuvant activity might be achieved without toxicity.

These studies document that HBsAg-NE is a safe and potent mucosal adjuvant for a novel hepatitis B vaccine. Its unique benefits include needle-free mucosal administration, induction of systemic immunity comparable with conventional vaccines, as well as mucosal and cellular immune responses that are not elicited by injected, aluminum-based hepatitis vaccines. The vaccine has stability that could improve its utility in the developing world. Given the ability to induce potent Th1 cellular immunity, this vaccine also may provide therapeutic benefit to patients with chronic HBV infection who lack cellular immune responses to adequately control the virus.

## Materials and Methods

### Adjuvant and antigen

Nanoemulsion (NE, W_80_5EC formulation) was supplied by NanoBio Corporation, Ann Arbor, MI. Nanoemulsion was manufactured by emulsification of cetyl pyridinium chloride (CPC, 1%), Tween 80 (5%) and ethanol (8%) in water with soybean oil (64%) using a high speed emulsifier, with resultant mean droplet size of less than 400 nm in diameter. W_80_5EC is formulated with surfactants and food substances that are ‘Generally Recognized as Safe’ (GRAS) by the FDA, and can be economically manufactured under Good Manufacturing Practices (GMP). The nanoemulsion is stable for at least 3 years at 25° C.

Recombinant HBs antigen *adw* serotype used for immunizations (HBsAg) was supplied by Human Biologicals Institute (Indian Immunologics, Ltd., Hyderabad, India). The antigen protein was purified from *Pichia pastoris* transfected with plasmid pPIC3K using standard methods according to Indian Immunologicals SOP and GMP procedures. HBsAg was dissolved in PBS (pH 7.03) and endotoxin level was determined to be <7.5 EU/20 μg of protein; below international standard of ≤30 EU/20 μg of protein.

### General reagents

Phosphate buffered saline (1×PBS and 10×PBS, pH 7.4) was purchased from Cellgro (Medtech, Inc). Deionized water was prepared using a Milli-Q® Ultrapure Water Purification system (Millipore, Billerica, MA). The bovine serum albumin (BSA) was purchased from Sigma. Alkaline phosphatase (AP) conjugated rabbit anti-mouse IgG (H&L), IgG1, IgG2a, IgG2b, IgG3, IgA (α chain specific), goat anti-rat IgG (H&L), and goat anti-guinea pig IgG (H&L) secondary antibodies were purchased from Rockland Immunochemicals, Inc.

### Particle sizing studies

HBsAg-NE formulations were prepared by vigorously mixing concentrated NE with HBsAg and PBS. Mixtures contained a final concentration of 0.5 mg/ml or 2.5 mg/ml of antigen mixed in 1%, 20%, or 40% (v/v) NE concentrations and normalized to 1×PBS.

The lipid-phase NE droplets were sized by quasi-elastic light scattering using an LS230 instrument (Beckmann-Coulter, Fullerton, CA) following manufacturer's protocols. In brief, between 10 μl and 30 μl of NE-antigen mixtures were diluted into a flow chamber containing 1 L of deionized water. Particle size distributions were calculated using number weighting, and statistics were generated from the average of three 60 second measurement cycles. Sample concentration was optimized based on PIDS obscuration, and PIDS data was included in the instrument's Fraunhofer model calculation.

### HBsAg analysis

The integrity of HBsAg protein was analyzed using sodium dodecylsulphate-polyacrylamide gel electrophoresis (SDS-PAGE) and Western blotting techniques. HBsAg was mixed in 20% NE at 0.5 mg/ml and 2.5 mg/ml concentrations. Aliquots of each of the HBsAg-NE mixtures were incubated at 4°C, 25°C and 40°C for up to 72 hrs. For PAGE analysis, the HBsAg samples were resuspended in 1% SDS, reduced with β-mercaptoethanol (BME, 2.5%) and boiled for 15 minutes. The electrophoresis was performed in duplicates using 0.5ug HBsAg, 4–12% Bis-Tris PAGE gels (Invitrogen), and MES SDS Running Buffer. One gel of each duplicate was stained using the SilverQuest Silver Staining Kit (Invitrogen). For Western blots, gels were transferred onto Immobilon-P PVDF membrane (Millipore) in NuPAGE transfer buffer according to Invitrogen's protocol. The membranes were blocked for 1 hr in 5% Milk/PBST and were probed with a polyclonal goat anti-HBsAg (Abcam). Alkaline phosphatase-(AP) conjugated anti-goat (Sigma) secondary antibodies were used with 1-Step NBT/BCIP AP substrate (Pierce) for protein detection.

### Zeta potential measurment

Zeta potential measurements were obtained using a NICOMP™ 380ZLS (PSS.NICOMP, Santa Barbara, CA). Samples containing 20% NE mixed with 2.5 mg/ml HBsAg were prepared by vigorously mixing concentrated NE and HBsAg. Test mixtures were diluted in either PBS or de-ionized water. Zeta potential was measured in 200×diluted samples at 25°C.

### Isothermal titration calorimetry

The interaction of the amphiphilic HBsAg with the lipid phase of NE was studied using an isothermal titration microcalorimeter (VP-ITC MicroCalorimeter, Microcal™). HBsAg solutions in PBS aliquots were prepared from concentrated stock and introduced into the calorimetric reaction and reference vessels (1.3 ml). Chambers were then gently agitated until temperature equilibrium with the surroundings was reached. Concentrated NE (50% wt) was diluted in PBS to 1% (v/v). After the sample vessel had reached the equilibrium conditions, the NE solution was added in discrete injections using a syringe, into the calorimetric reaction vessel under continuous stirring (either 30°C or 40°C). The experimentally observed change of energy corresponding to a given injection of NE was measured and plotted using software (Origin 7SR4 v. 7, Origin Lab Corp., Northhampton, MA). The change in heat capacity of binding (ΔCp) was calculated using the following equation: ΔCp = (ΔH°_T2_-ΔH°_T1_)/T2-T1 where ΔH is calculated enthalpy and T is vessel temperature [71].

### Preparation of HBsAg-NE vaccine

HBsAg-NE formulations were prepared 30 to 60 minutes prior to immunization by vigorously mixing HBsAg protein solution with concentrated NE using PBS as diluent. For intranasal immunizations HBsAg-NE doses ranged from 1 μg to 40 μg HBsAg mixed with 5% to 40% NE. For intramuscular immunizations with the HBsAg/aluminum hydroxide vaccine (HBsAg-Alu), antigen was adsorped onto 0.5 mg/ml aluminium hydroxide (Sigma) following the adsorption procedure described in [72] to obtain formulation similar to that of Engerix® (GlaxoSmithKline).

### Animals

Pathogen-free, outbred CD-1 mice (females 6–8 weeks old), inbred BALB/c mice (females 6–8 weeks old), and Hartley guinea pigs (females 10–11 weeks old) were purchased from Charles River Laboratories. Pathogen free Sprague Dawley rats (females 7–8 weeks old) and specific pathogen free (SPF) purpose-bred American standard beagles (females, 6 month old) were obtained from Harlan and Covance, respectively. Animals used in these studies were housed in SPF conditions with food and water available ad libitum in accordance to the standards of the American Association for Accreditation of Laboratory Animal Care. Mice were housed with 5 to a cage. Rats and guinea pigs were housed 3 to a cage. Dogs were housed in floor pens with soft bedding and in a rotating group setting. Daily exercise was provided as enrichment. All procedures performed on animals within this study were conducted in accordance with and by approval of the University of Michigan University Committee on Use and Care of Animals (UCUCA).

### Immunization procedures

CD-1 mice (*n = 5* per group for immunogenicity studies, *n* = 10 per group for stability studies) were vaccinated with two administrations of HBsAg-NE vaccine six weeks apart. Both intranasal (i.n.) and intramuscular (i.m.) immunizations were performed in mice anaesthetized with isoflurane using IMPAC 6® anesthesia delivery system. For i.n. administration, animals were held in a supine position and 8 μl (4 μl/nare) of HBsAg-NE vaccine was administered slowly to the nares using a micropipette tip. For i.m. immunization, the 50 μl of HBsAg-Alu vaccine was injected into apaxial muscle. Rats, and guinea pigs (*n* = 5 per group) were also manually restrained in a supine position and 100 μl (50 μl/nare) of HBsAg-NE vaccine was administered slowly to the nares using a micropipette tip.

### Blood, bronchioalveolar lavage, and splenocyte collection

Blood samples were obtained from the saphenous vein in mice, rats, and guinea pigs and from the superficial cephalic vein in dogs at various time points during the course of the experiments. The terminal murine sample was obtained by cardiac puncture post-euthanasia. Serum was separated from whole blood by centrifugation at 1500×g for 5 minutes after allowing coagulation for 30 to 60 minutes at room temperature. Serum samples were stored at −20°C until analyzed.

Bronchioalveolar lavage (BAL) fluid was obtained from mice euthanized by an overdose of isoflurane. A 22 gauge catheter (Angiocath, B-D) attached to a syringe was inserted into the distal trachea. The lungs were infused twice with 0.5 ml of PBS containing 10 μM DTT and 0.5 mg/ml aprotinin and approximately 1 ml of aspirate was recovered. BAL samples were stored at −20°C until analyzed.

At the time of euthanasia, spleens were harvested from mice and mechanically disrupted to obtain single-cell splenocyte suspension in PBS, which was used for in vitro determination of cytokine response. Red blood cells were removed by lysis with ACK buffer (150 mM NH_4_Cl, 10 mM KHCO_3_, 0.1 mM Na_2_EDTA), and the remaining cells were washed twice in PBS. For the cytokine expression assays, splenocytes were resuspended in RPMI 1640 medium supplemented with 2% FBS, 200 nM L-glutamine, and penicillin/streptomycin (100 U/ml and 100 μg/ml).

### Determination of IgG and IgA antibodies in serum and BAL fluid

Mouse, rat, and guinea pig anti-HBsAg specific IgG and mouse anti-HBsAg specific IgA levels were determined by ELISA. Microtiter plates (NUNC) were coated with 5 μg/ml (100 μl) of HBsAg in a coating buffer (50 mM sodium carbonate, 50 mM sodium bicarbonate, pH 9.6) and incubated overnight at 4°C. The protein solution was removed and plates were incubated with blocking buffer (PBS with 1% dry milk) for 30 minutes at 37°C. After the blocking solution was aspirated, the plates were used immediately or stored sealed at 4°C until needed. For antibody detection, serum and BAL samples were serially diluted in 0.1% BSA in PBS. The 100 μl/well aliquots were incubated in HBsAg coated plates for 1 hour at 37°C. Plates were washed three times with PBS containing 0.05% Tween 20, followed by 1 hour incubation with either species specific anti-IgG or IgA alkaline phosphatase (AP)-conjugated antibodies, then washed three times and incubated with AP substrate Sigma Fast™ (Sigma). The colorimetric reaction was stopped with 1 N NaOH according to the manufacturer's protocol, and optical density (OD) measured using a Spectra Max 340 ELISA reader (Molecular Devices, Sunnyvale, CA) at 405 nm and the reference wavelength of 690 nm. The antibody concentrations are presented as endpoint titers defined as the reciprocal of the highest serum dilution producing an OD above cutoff value. The cutoff value is determined as OD of the corresponding dilution of control sera+2 (standard deviations) and plate background [73], [74]. Normalization of IgG was performed at UMHHC diagnostic laboratory using an ADVIA Centaur anti-HBsAg assay.

### Determination of IgG avidity

The avidity index (AI) was determined by ELISA using mouse serum as described by Vermont et al. [75] with minor modifications. Sodium thiocyanate (NaSCN) was used for dissociation of low avidity antibody-antigen binding. Optimal assay conditions for determination of AI were established in an ELISA assay using 0 M to 3 M range of NaSCN concentrations. We have identified that incubation with 1.5 M NaSCN solution resulted in reduction of antibody binding that was discriminating between serum samples. In each assay, serial dilutions of immune serum were incubated with HBsAg as described above for standard ELISA. To differentiate antibody binding, the wells were incubated with either PBS or with 1.5 M NaSCN at room temperature for 15 minutes. Subsequently wells were washed three times and incubated with anti-mouse IgG AP-conjugate as described above. The AI was calculated as percentage of antibody titer which remained bound to antigen after incubation with NaSCN in comparison to the standard ELISA protocol.

### Luminex analysis of cytokine expression

Freshly isolated mouse murine splenocytes were seeded at 4×10^6^ cells/ml (RPMI 1640, 2% FBS) and incubated with HBsAg (5 μg/ml) or control PHA-P mitogen (2 μg/ml) for 72 hours. Serum and cell culture supernatants were harvested and analyzed for the presence of cytokines. These assays were performed using Luminex®Multiplex21 multi-analyte profiling beads (Luminex Corporation, Austin, TX), according to the manufacturer's instructions.

### Analyses of thermostability of HBsAg-NE

For vaccine thermostability studies, the formulation was made by vigorously mixing HBsAg and NE to achieve a dose of 2.5 mg/ml recombinant protein in 20% NE and a final buffered solution of 1× PBS. The vaccine was then aliquoted into sterile glass vials with Teflon-coated caps (Wheaton) and stored at either 4±2°C, 25±2°C or 40±2°C. Temperatures were monitored for the period of the study by Lufft OPUS10 thermographs (PalmerWahl). At time points of 6 weeks, 12 weeks (3 months), 24 weeks (6 months) and 52 weeks (1 year), an aliquot was withdrawn and used for in vitro as well as in vivo analyses. For in vitro analyses 0.5 μg of antigen contained in vaccine product was electrophoresed per lane and detected by silver staining and Western blotting (as described above); NE particle size was also determined (as described above). In vivo immunogenicity studies were done by intranasal vaccinations (primed at 0 and boosted at 6 weeks) of about 8 week old female CD-1 mice (n = 10 per group) and testing serum IgG titers at 2, 3, 5, 8, 10 and 12 weeks as described above.

### Comprehensive toxicity assessments

Acute and (sub) chronic toxicity responses to either NE or HBsAg-NE were assessed in mice, rats, guinea pigs, and dogs. We have evaluated numerous species in order to minimize the effects of animal model biasing. The end points of the study were histopathological evaluation of exposed tissues and of highly perfused organs. Metabolic changes were also measured using serum biochemical profile analysis.

The clinical status of each animal including the nasal cavity, body weight, body temperature, and food consumption was assessed throughout the study. Mice were non-surgically implanted with programmable temperature transponders (IPTT-3000, Bio Medic Data Systems, Inc.) for non-invasive subcutaneous temperature measurement with a handheld portable scanner (DAS-6002, Bio Medic Data Systems, Inc.). Euthanasia by isoflurane asphyxiation was performed in mice whereas rats and guinea pigs were euthanized by barbiturate overdose. A complete necropsy, which included the gross pathological examination of the external surface of the body, all orifices, and the cranial thoracic and abdominal cavities and their contents, was performed on all rodent species at the time of death. Vaccine exposed tissues and highly perfused organs including the sinus cavity, lungs, esophagus, trachea, brain, heart, liver, kidneys, spleen, stomach, intestines, pancreas, and adrenals were collected and immediately fixed in 10% buffered formalin (Fisher Scientific).

In order to assess safety and tolerability of the adjuvant, NE was delivered to dogs using a wide angle nasal sprayer pump (Pfeiffer 62602, 415 screw enclosure). The containers used were Saint Gobain Desqueres 5-mL U-Save™ Type 1 amber glass bottles with a 415 neck finish. The dose volume for the sprayer pump was 100 μl. Dogs received either 200 μl (100 μ/nare) or 400 μl (200 μl/nare) administered every 14 days for a total of 3 doses as outlined in **Table 2**. Rostral nasal sinus punch biopsy samples were collected 24 hours following the final treatment. For the biopsy procedure, dogs were anesthetized with ketamine/diazepam/butorphanol (10 mg/kg, 0.5 mg/kg, 3 mg/kg) and maintained on 2.5% isoflurane after endotracheal intubation. The anterior sinus cavity and external nares were sterilely prepared. A sterile dermal punch biopsy instrument (Miltex, 4 mm) was introduced approximately 1.5 cm into ventral portion of the anterior sinus cavity. Hemostasis was achieved using 4-0 PDS suture material. Tissues obtained for biopsy were immediately fixed in 10% buffered formalin (Fisher Scientific). Butorphanol (3 mg/kg) administration was continued every 8 hours for three days following the biopsy procedure for analgesic management.

### Histopathological analysis

Harvested tissues were fixed in 10% formalin solution for at least 24 hours. Sinus tissues including bone were decalcified for 48 hours using Cal-EX™ II (Fisher Scientific) prior to trimming and embedding in paraffin. For mice, rats, and guinea pigs, four standard cross sections of the nasal passages including the brain were taken [76]. Tissue blocks were processed in xylene and paraffin embedded for multi-sections and slide preparation. Routine hematoxylin and eosin (H&E) staining of each slide was carried out and blindly examined by a veterinary pathologist. Histopathological lesions were scored on a histological grading scale ranging from 0 to 10 based on severity and distribution.

The histopathology of the nasal cavity was scored using very strict criteria. Any finding other than pristine was given a positive score. A single small focus of accumulation of amorphous material and/or the presence of any cell damage no matter how slight was scored as +1 (**Figure 9C**). More than one focus of accumulation of material and/or cell damage was scored as +2 (**Figure 9D**). More than 3 foci of accumulation of material and/or cell damage or multiple locally extensive areas of pathology were scored as +3. The lesions graded as +4 to +6 were associated with increasing severity and more extensive distribution of lesions including the presence of lesions in more than one section. These lesions could be associated with morbidity. The +7 and above had increasing degrees of inflammation. Mortality would be given a score of +10.

### Hematological and serum biochemical profile analysis

Whole blood samples were collected from rats and guinea pigs 2 weeks following the final vaccine dose. Dogs were phlebotomized every 14 days and at the study termination at day 43. A portion of the blood was placed in Vacutainer™ tubes containing EDTA (BD) and a portion was placed in serum separator Vacutainer™ tubes (BD). Anti-coagulated blood was processed to determine hematological parameters (lymphoyctes, monocytes, eosinophils, basophils, red blood cells, hemoglobin , hematocrit, mean corpuscular hemoglobin, mean corpuscular volume, mean corpuscular hemoglobin concentration, and platelets) in a HEMAVET® 950 hematology analyzer (Drew Scientific, Inc., Oxford, CT) in accordance to manufacturer's recommendation. Hematological data was compared to species laboratory reference values as established by the Animal Diagnostic Laboratory at the University of Michigan.

Serum samples were analyzed using a VetTest Chemistry Analyzer® (Idexx, Westbrook, Maine). A complete chemistry panel including albumin, alkaline phosphatase, alanine aminotransferase, amylase, aspartate aminotransferase, total calcium, total cholesterol, creatinine, glucose, phosphorous, total bilirubin, total protein, blood urea nitrogen, sodium, potassium, chloride, globulin, and creatine kinase was performed. Biochemical data was compared to species laboratory reference values as established by the Animal Diagnostic Laboratory at the University of Michigan.

### Statistical Analysis

Results are expressed as mean±standard error of the mean (SEM) or±standard deviation (SD). Statistical significance was determined by ANOVA (analysis of variance) using the Student *t* and Fisher exact tests or a Bonferroni's Multiple comparison analysis. The analyses were done with 95% confidence limits and two-tailed tests. A *p value*<0.05 was considered to be statistically significant.
